# Individual heart failure patient variability in nocturnal hypoxia and arrhythmias

**DOI:** 10.1097/MD.0000000000040083

**Published:** 2024-10-11

**Authors:** Cubby L. Gardner, Harry B. Burke

**Affiliations:** a Daniel K. Inouye Graduate School of Nursing, Uniformed Services University, Bethesda, MD; b F. Edward Hébert School of Medicine; Uniformed Services University, Bethesda, MD.

**Keywords:** heart failure, heart rate, home environment, oxygen saturation, wearable devices

## Abstract

Traditional heart failure research often uses daytime population parameter estimates to assess hypoxia and arrhythmias. This approach might not accurately represent heart failure patients as nighttime cardiac behaviors offer crucial insights into their health, especially regarding oxygen levels and heart rhythms. We conducted a prospective study on nocturnal oxygen saturation and heart rate in home-dwelling heart failure patients over 6 nights. Patients were recruited from the Walter Reed National Military Medical Center heart failure clinic. Criteria included a clinical diagnosis of heart failure, a New York Heart Association (NYHA) classification of I to III, ages between 21 to 90, cognitive intactness, capability to use the wearable device, and willingness to use the device for 6 consecutive nights. Average oxygen saturation was 92% with individual readings ranging from 40% to 100%. The mean heart rate was 72 beats per minute (bpm), but individual rates ranged from 18 bpm to a high of 296 bpm. A significant drop in oxygen levels and sleep arrhythmias were consistently observed among participants. Heart failure patients demonstrate notable and variable desaturations and arrhythmias across multiple nights. A single-night sleep study or a 24-hour heart rate monitor may not comprehensively depict patients’ oxygenation and heart rate irregularities. Our research highlights wearable devices’ potency in medical research for capturing essential nocturnal data. In only 6 nights, we gleaned invaluable clinical insights for optimizing patient care. This study is pioneering, being the first to intensively examine nighttime oxygen levels and heart rates in home-based heart failure patients.

## 1. Introduction

Heart failure is a progressive condition characterized by symptoms of shortness of breath, a racing heart, and fatigue.^[1]^ It causes one-ninth of all deaths.^[2]^ Its prevalence is expected to increase to 8.5% of 65 to 70-year-olds^[3,4]^ and its cost is expected to increase to $70 billion by 2030.^[5]^ Many heart failure patients experience acute decompensations, especially at night,^[6]^ and require frequent emergency department visits,^[7–9]^ hospitalizations,^[5,10,11]^ and readmissions.^[12,13]^ A recent U.S. Department of Veterans Affairs readmission study found their 30-day and 1-year mortality to be 5.6% and 25.0%, respectively.^[12]^

Historically, heart failure research has assessed hypoxia and arrhythmias using daytime population parameter estimates. This may not be an accurate characterization of heart failure patients for 2 reasons. First, there can be substantial intra- and inter-patient variability within and across days. Second, it does not consider the fact that heart failure patients exhibit clinically important hypoxic and dysrhythmic episodes at night.

The routine management of heart failure patients occurs during daytime office visits where a physical examination is conducted, patients’ oxygenation is assessed, heart rate and blood pressure are measured, and patients are asked to remember how they felt during their activities of daily living.^[14]^ Unfortunately, because of physiological, functional, psychological, and cognitive declines, many heart failure patients find it difficult to monitor and manage their clinical condition^[10,11]^ and their historical self-reports are not always accurate and reliable.^[11,15]^

One approach to obtaining accurate and reliable patient data is wearable devices which record clinical data in patients’ homes and provide ecologically valid information to clinicians.^[16]^ Wearable devices have gained widespread clinical acceptance^[16–18]^ and are reimbursed by the Centers for Medicare & Medicaid Services.^[14,19]^ They can monitor patients’ heart rate^[20,21]^ and oxygen saturation,^[22]^ and they can be used to manage medications.^[23]^ They are also being used to predict emergency department visits, readmissions, and death.^[24–26]^ In addition, wearable devices are being incorporated into clinical trials as part of the intervention assessment.^[27]^ Studies have demonstrated that wearable devices are effective in reducing hospitalizations and improving the quality of life of heart failure patients.^[28]^ Moreover, the use of wearable devices has been shown to be cost-effective, potentially leading to reduced healthcare costs.^[29]^

Our ability to obtain accurate and reliable clinical data while patients are at home using wearable devices has opened a window to their lives. For example, it allows us to objectively assess patients’ activity levels during the day.^[30]^ Although wearable devices have been used at night,^[16]^ and have measured sleep quality,^[31]^ little is known about heart failure patients’ clinical status over successive nights. We have previously shown that heart failure patients can reliably use wearable devices at home every night for 6 nights, and we have also shown that the information collected by these devices can be displayed in a way that clinicians find useful in their management of heart failure patients.^[32,33]^ What we have not shown is that the collected and displayed information is clinically actionable. We hypothesize that a that heart failure patients demonstrate clinically important night-to-night variability in their oxygen saturation and heart rate, variability that would not be detected by single-night sleep studies or 24-hour heart rate monitoring. Wearable devices can be used by heart failure patients at home over 6 nights to capture clinically important and ecologically valid desaturations and arrhythmias. To our knowledge, this is the first ecologically valid moment-by-moment synchronous nocturnal study of oxygen saturations and heart rates at night for 6 nights in home-dwelling heart failure patients.

## 2. Methods

This prospective study assessed the nocturnal oxygen saturation and heart rate status of home dwelling heart failure patients over 6 nights. Patients were recruited from the Walter Reed National Military Medical Center heart failure clinic. The study patients were retirees and family members of retirees. This population, and its care, has been shown to be similar to that of the general population.^[34–37]^ Patients were treated in accordance with American Heart Association heart failure guidelines.^[7]^ The Walter Reed National Military Medical Center institutional review board approved this study.

Recruitment, consenting, and study instructions were done during routinely scheduled outpatient visits. In accordance with the study protocol, consecutive patients waiting in an examination room prior to their scheduled clinical encounter were given information about the study, they were shown the wearable device, and its functioning was explained to them. Participation was requested and, if agreed to, patients read and signed the informed consent form. Inclusion criteria were a clinical diagnosis of heart failure, a New York Heart Association (NYHA) classification of I to III, 21 to 90 years of age, cognitively intact, physically able to operate the wearable device, and they agreed to use the device for all 6 nights of the study. The decision to use a 6-night duration for this study was based on a combination of factors, including previous literature, feasibility and participant adherence. We aimed to strike a balance between capturing meaningful, clinically actionable data and minimizing participant burden, which can affect compliance in longer studies.

Patients were given the Nonin WristOx2 model 3150 pulse oximeters, a wrist-worn, battery-operated device that they wore at night for 6 nights. Powered by 2 fresh 1.5V alkaline AAA batteries, the ample battery life permitted collection of 48 hours of data at 1-second intervals; sufficient for 6 nights of data. The device was set to automatically record only when the flexible probe was placed on the patient’s finger. Patients put the device on when going to bed at night, wore it continuously overnight, and took it off when they got out of bed in the morning.

Patients were contacted by telephone after the first night to answer questions. Seven days after enrollment, patients returned to the heart failure clinic with their study devices, and they completed a usability assessment. None of the patients reported any problems using the devices and there were no device failures. The device-collected data were downloaded using device-specific software, cleaned using the R statistical language, and uploaded into the study’s MySQL study database.

Descriptive statistics were calculated. For each patient each night, the mean, standard deviation (SD), range, and coefficient of variation (CV) were calculated for: (1) blood oxygen saturation, (2) bradycardia, heart rate below 60 beats per minute, and (3) tachycardia, heart rate above 100 beats per minute. Differences in continuous variables were assessed by the Student *t* test and differences in categorical variables was assessed by the Chi-square test. For each patient, a one-way repeated measures analysis of variance (ANOVA) assessed for oxygen saturation across the 6 nights, bradycardia across the 6 nights, and tachycardia across the 6 nights.

To test night-to-night variability of oxygen saturation and heart rate within patients a one-way ANOVA was constructed for each patient to assess their values by night. A similar one-way ANOVA was constructed for each patient to assess heart rates <60 beats per minute by night and to assess heart rate above 100 beats per minute by night. To assess the effect, if any, of continuous positive airway pressure (CPAP), we separated the study cohort into a CPAP + (patients receiving CPAP at night) and CPAP ‐ (patients not receiving CPAP at night) cohorts. We repeated the above analyses for each of these cohorts.

Kaplan–Meier plots were constructed for: (1) the percentage of patients who experienced desaturation episodes (oxygen saturation at or below 94%) lasting 10 to 59 seconds, 60 to 299 seconds, and 300 and greater seconds (mutually exclusive categories); (2) the percentage of patients who experienced bradycardic episodes lasting 10 to 59 seconds, 60 to 299 seconds, and 300 and greater seconds (mutually exclusive categories); and (3) the percentage of patients who experienced tachycardic episodes lasting 10 to 59 seconds, 60 to 299 seconds, and 300 and greater seconds (mutually exclusive categories). Significant differences in the Kaplan–Meier plots were assessed by the log-rank test. For all patients over all nights, a correlation plot for oxygen saturation <94% and the associated heart rate reading was constructed with linear regression and *R*^2^ for the association. All analyses were performed in R (R Project) https://www.r-project.org/.

## 3. Results

Twenty-six heart failure patients participated in the study (Table 1). Their mean age was 69 years (SD, 12 years), 69% were men, and 62% were African American. NYHA classifications frequencies were Class I, 7 patients; Class II, 14 patients; and Class III, 5 patients. Twenty-two of the 26 patients reported co-morbid conditions and 20 of 26 patients had >1 co-morbid condition. Ten patients had implantable cardiac devices, one patient was prescribed nocturnal oxygen therapy, and 7 were prescribed CPAP. Comparing patients with and without implanted cardiac devices, there were no significant differences in age, gender, marital status, NYHA class, or co-morbid conditions. Seven patients had sleep apnea, 4 of whom were receiving CPAP. Seven patients were prescribed CPAP therapy, 4 of whom had sleep apnea. Comparing patients with and without CPAP, there were no significant differences in age or the distribution of gender, marital status, NYHA class, or co-morbid conditions.

**Table 1:** Patient demographics and comorbid conditions.

Demographics	
Number of subjects	26
Age, mean ± SD	69 ± 12
Gender, N (%)	
Male	18 (69%)
Female	8 (31%)
Race/ethnicity, N (%)	
African American	16 (62%)
Caucasian	10 (38%)
NYHA classification, N (%)	
I	7 (27%)
II	14 (54%)
III	5 (19%)
Implanted cardiac device, N (%)	
CRT	6 (23%)
ICD	2 (8%)
PPM	2 (8%)
No device	16 (61%)
Prescribed nocturnal O_2_	1 (4%)
Prescribed CPAP	7 (27%)

Co-morbid conditions	Frequency
Hypertension	21
Hyperlipoproteinemia	13
Diabetes	11
Arthritis	10
Coronary artery disease	8
Chronic obstructive pulmonary disease	8
Obstructive sleep apnea	7
Renal disease	5
Chronic pain	4
Depression/anxiety	3
Chronic fatigue syndrome	2
Chronic headache	2

Across all patients for all nights, the mean oxygen saturation was 92% (SD, 4), the range was 40% to 100%, the mean minimum oxygen saturation was 69 (SD, 12), and the mean maximum blood oxygen saturation was 99 (SD, 1). The mean heart rate was 72 beats per minute (bpm) (SD, 12), the range was 18 to 296 bpm, the mean minimum heart rate was 38 bpm (SD, 13), and the mean maximum heart rate was 183 bpm (SD, 65).

Table 2 shows the individual mean, standard deviation, and range of blood oxygen saturation for each patient each night stratified by CPAP status. All the patients experienced desaturations and all the patients exhibited significant variability within each night (*P* < .0001) and across the 6 nights (*P* < .0001). The grand means of the CPAP + cohort (91.37, SD 0.23) and the CPAP ‐ cohort (92.27, SD 0.37) were significantly different (*P* = .0008). Table S1, Supplemental Digital Content, http://links.lww.com/MD/N730 shows the coefficient of variation for the desaturations for each patient each night and across nights.

**Table 2:** Mean (standard deviation) [range] of blood oxygen saturation for each patient across the 6 nights. There was a significant effect of night on blood oxygen saturation across nights (*P* < .0001) for all patients.

Patient	Night 1	Night 2	Night 3	Night 4	Night 5	Night 6
CPAP+						
D	90.4 (2.61) [85, 96]	89.1 (2.45) [80, 98]	89.1 (2.44) [80, 95]	89.0 (3.08) [79, 95]	89.5 (2.45) [55, 98]	89.5 (3.08) [78, 99]
E	89.5 (1.79) [79, 94]	91.1 (2.06) [77, 97]	90.5 (2.29) [79, 98]	90.8 (1.45) [84, 95]	90.2 (1.83) [80, 96]	90.2 (2.14) [78, 97]
F	92.0 (2.20) [70, 98]	92.3 (2.17) [82, 98]	93.0 (1.75) [79, 98]	93.3 (1.83) [82, 99]	91.2 (3.10) [76, 98]	93.7 (1.71) [84, 98]
I	91.9 (2.38) [77, 100]	90.2 (2.23) [79, 96]	91.0 (2.04) [78, 97]	91.7 (1.54) [81, 98]	92.0 (2.09) [76, 98]	93.7 (1.65) [82, 99]
S	88.8 (2.04) [80, 96]	90.2 (1.94) [82, 97]	88.0 (3.08) [78, 98]	90.5 (2.54) [82, 99]	91.2 (3.06) [62, 99]	88.5 (2.04) [63, 96]
U	93.9 (1.45) [87, 99]	92.2 (1.75) [83, 99]	92.4 (1.75) [82, 98]	93.8 (1.80) [88, 99]	92.0 (1.85) [80, 98]	91.5 (1.87) [86, 98]
X	93.7 (1.11) [90, 98]	94.1 (1.13) [89, 99]	93.3 (0.95) [79, 98]	92.7 (1.74) [85, 98]	94.0 (1.46) [82, 98]	92.2 (1.74) [83, 97]
**Mean**	**91.5 (1.94) [81, 97]**	**91.3 (1.96) [82, 98]**	**91.0 (2.04) [79, 97]**	**91.7 (2.00) [83, 98]**	**91.4 (2.26) [73, 98]**	**91.3 (2.03) [79, 98]**
CPAP‐						
A	92.8 (0.99) [84, 97]	93.9 (1.15) [89, 99]	93.8 (1.10) [88, 98]	93.6 (1.36) [87, 98]	93.6 (1.08) [90, 98]	94.3 (1.12) [90, 99]
B	91.4 (2.07) [82, 98]	94.3 (1.87) [86, 100]	93.5 (1.86) [54, 99]	94.0 (1.63) [86, 100]	93.8 (1.81) [84, 98]	93.6 (1.42) [80, 98]
C	88.2 (2.36) [74, 96]	88.7 (2.94) [72, 99]	88.2 (3.11) [70, 99]	89.2 (2.76) [74, 97]	89.4 (2.48) [74, 98]	89.5 (3.37) [74, 99]
G	92.7 (1.48) [83, 99]	93.9 (1.46) [66, 100]	92.9 (1.42) [83, 99]	93.6 (1.36) [84, 99]	93.0 (1.49) [89, 98]	94.1 (1.10) [90, 98]
H	93.3 (1.77) [84, 99]	93.3 (1.68) [85, 100]	93.1 (1.78) [81, 99]	93.1 (1.74) [82, 99]	93.9 (1.61) [84, 99]	93.2 (1.51) [83, 98]
J	82.2 (4.13) [64, 94]	83.5 (5.76) [65, 98]	81.9 (3.74) [61, 95]	81.4 (3.89) [63, 95]	82.7 (3.39) [68, 93]	82.5 (4.51) [67, 100]
K	89.2 (3.18) [80, 98]	91.7 (4.04) [80, 100]	90.1 (3.45) [80, 97]	90.5 (2.92) [83, 97]	91.7 (2.47) [84, 97]	90.8 (2.57) [81, 96]
L	88.1 (4.24) [66, 100]	93.1 (3.86) [59, 99]	95.4 (4.17) [45, 100]	93.6 (3.23) [74, 99]	93.4 (3.30) [77, 99]	92.7 (3.29) [68, 100]
M	94.0 (1.11) [84, 98]	94.8 (1.41) [85, 100]	93.8 (1.24) [81, 98]	94.2 (1.10) [86, 98]	93.9 (1.42) [83, 99]	94.3 (1.31) [85, 99]
N	89.0 (4.26) [75, 98]	90.5 (3.58) [76, 99]	90.5 (3.68) [75, 100]	90.6 (2.89) [78, 98]	92.1 (4.04) [71, 100]	94.2 (1.88) [82, 99]
O	93.9 (1.80) [83, 98]	94.0 (1.59) [82, 98]	92.9 (1.54) [82, 97]	95.7 (1.50) [88, 100]	93.4 (1.89) [81, 99]	93.5 (1.86) [80, 98]
P	92.1 (2.55) [51, 99]	90.9 (4.89) [43, 100]	90.6 (3.28) [54, 100]	92.1 (4.67) [40, 99]	94.9 (2.62) [89, 100]	91.8 (5.99) [41, 99]
Q	94.3 (2.87) [82, 99]	94.6 (2.35) [83, 99]	93.3 (3.59) [79, 99]	94.3 (2.04) [82, 99]	93.7 (2.61) [52, 99]	93.1 (2.87) [78, 99]
R	95.0 (1.75) [83, 100]	94.3 (1.41) [82, 100]	94.8 (1.65) [86, 100]	94.6 (1.93) [85, 100]	93.8 (2.06) [79, 99]	95.4 (2.09) [79, 100]
T	92.4 (1.90) [84, 98]	91.3 (1.96) [83, 98]	93.3 (1.63) [80, 99]	91.1 (1.53) [84, 97]	91.3 (1.70) [86, 98]	93.0 (1.85) [74, 98]
V	94.6 (1.32) [86, 99]	94.9 (1.23) [87, 99]	94.7 (1.09) [88, 99]	94.7 (1.27) [86, 99]	93.8 (1.11) [86, 97]	94.9 (1.52) [68, 99]
W	93.2 (2.93) [73, 99]	92.1 (2.91) [71, 98]	93.2 (1.97) [76, 98]	94.3 (1.98) [70, 98]	95.4 (3.10) [82, 100]	93.6 (2.49) [84, 100]
Y	91.5 (3.58) [69, 99]	91.4 (3.36) [73, 99]	91.0 (1.88) [82, 97]	91.6 (1.71) [83, 97]	91.1 (1.86) [79, 98]	89.5 (2.03) [78, 97]
Z	92.0 (2.74) [76, 99]	94.0 (2.70) [80, 99]	93.3 (2.32) [82, 100]	93.4 (2.42) [83, 98]	95.4 (3.10) [82, 100]	93.6 (2.49) [84, 100]
**Mean**	**91.6 (2.48) [77, 98]**	**92.4 (2.64) [77, 99]**	**92.1 (2.34) [75, 99]**	**92.4 (2.21) [79, 98]**	**92.6 (2.28) [79, 98]**	**92.5 (2.36) [77, 99]**

Table 3 shows the individual mean, standard deviation, and range of bradycardia for each patient each night stratified by CPAP status. All the patients experienced bradycardia at night. Twenty-four patients’ bradycardia exhibited significant variability within each night (*P*-values varied from <.05 to <.001) and 21 patients demonstrated significant variability across the 6 nights (*P* < .0001). The grand means of the CPAP + cohort (53.27, SD 2.66) and the CPAP ‐ cohort (55.07, SD 0.62) were not significantly different (*P* = .16). Table S2, Supplemental Digital Content, http://links.lww.com/MD/N730 shows the coefficient of variation for the bradycardia for each patient each night and across nights.

**Table 3:** Mean (standard deviation) [range] of bradycardia for each patient who exhibited bradycardia across the 6 nights, blank spaces indicate nights where no heart rate value below 60 beats per minute was recorded.

Patient	Night 1	Night 2	Night 3	Night 4	Night 5	Night 6	
CPAP+							
D	56 (1.25) [55, 59]	–	–	34.7 (8.2) [32, 58]	42.2 (7.28) [30, 56]	48.5 (9.15) [31, 58]	***
E	–	–	48 (9.17) [38, 56]	–	57 (NA) [57, 57]	57 (NA) [57, 57]	
F	54.3 (2.26) [48, 59]	54.8 (2.04) [31, 59]	53.8 (2.74) [18, 59]	54 (2.81) [40, 59]	52.8 (3.06) [33, 59]	51.4 (3.87) [44, 59]	***
I	–	–	–	51 (0) [51, 51]	43.6 (10.1) [33, 57]	43.2 (10.5) [38, 59]	
S	48.6 (5.56) [40, 59]	54.9 (3.3) [49, 59]	55.4 (2.66) [50, 59]	53.5 (4.07) [45, 59]	52.8 (6.39) [43, 59]	52.9 (3.51) [48, 58]	***
U	58.3 (1.22) [54, 59]	59 (0) [59, 59]	59 (NA) [59, 59]		47.6 (13.67) [31, 58]	54.9 (2.59) [52, 59]	***
X	57.4 (1.71) [47, 59]	58.1 (1.12) [54, 59]	58.6 (0.6) [57, 59]	57.7 (1.46) [50, 59]	57.4 (1.89) [48, 59]	58.1 (1.95) [43, 59]	***
**Mean**	**54.9 (2.40) [49, 59]**	**56.7 (1.62) [48, 59]**	**55.0 (3.79) [44, 58]**	**50.2 (3.31) [44, 57]**	**50.5 (7.06) [39, 58]**	**52.3 (5.26) [45, 58]**	
CPAP‐							
A	–	58.3 (0.98) [54, 59]	–	56.8 (2.94) [45, 59]	55.5 (3.87) [42, 59]	58.6 (0.69) [55, 59]	***
B	58.7 (0.47) [58, 59]	57.4 (1.6) [51, 59]	57.4 (1.56) [52, 59]	58.3 (0.84) [56, 59]	55.7 (3.34) [42, 59]	58.1 (1.24) [55, 59]	***
C	–	56 (NA) [56, 56]	–	–	30 (NA) [30, 30]	39.6 (6.91) [26, 46]	*
G	53.5 (6.63) [33, 59]	58.7 (0.89) [50, 59]	57.6 (2.95) [35, 59]	57.6 (3.51) [33, 59]	57.9 (2.8) [34, 59]	58.8 (0.59) [50, 59]	***
H	–	–	–	–	–	–	
J	46 (NA) [46, 46]	–	41 (8.57) [20, 59]	55.6 (3.69) [43, 59]	55.3 (4.32) [30, 59]	55.2 (6.94) [39, 59]	***
K	58.3 (1.32) [51, 59]	57.9 (1.54) [54, 59]	57.6 (1.65) [47, 59]	58.1 (1.05) [55, 59]	57.6 (2.35) [49, 59]	55.5 (3.37) [43, 59]	***
L	56.8 (2.18) [43, 59]	55.5 (5.47) [28, 59]	55.9 (2.75) [35, 59]	56.6 (2.44) [41, 59]	57 (2.27) [32, 59]	56.7 (2.55) [37, 59]	***
M	59 (0.19) [58, 59]	57.9 (0.9) [57, 59]	58 (1.31) [53, 59]	57.9 (1.11) [56, 59]	57.8 (1.43) [53, 59]	57.4 (2.11) [48, 59]	***
N	59 (0) [59, 59]	53 (6.09) [38, 59]	57.3 (2.45) [48, 59]	51.1 (5.21) [39, 59]	46.6 (7.63) [37, 59]		***
O	58.1 (0.88) [56, 59]	58 (0) [58, 58]	–	54.9 (4.63) [45, 59]	57.6 (1.35) [55, 59]	57.8 (1.1) [57, 59]	*
P	–	–	–	–	–	–	
Q	55 (0) [55, 55]	–	55.5 (2.87) [50, 59]	–	–	–	
R	56.3 (1.55) [52, 59]	55.7 (1.42) [51, 59]	56.1 (1.95) [50, 59]	55.8 (1.54) [51, 59]	56.1 (1.68) [50, 59]	56.7 (1.39) [52, 59]	***
T	58.8 (0.51) [56, 59]	58.6 (0.88) [53, 59]	58.1 (3.87) [22, 59]	57.8 (3.87) [37, 59]	58 (1.55) [50, 59]	58.6 (0.7) [49, 59]	***
V	58.1 (1.34) [48, 59]	56.9 (2.1) [43, 59]	58 (1.57) [49, 59]	58.2 (1.04) [53, 59]	58.1 (0.95) [55, 59]	58 (1.57) [47, 59]	***
W	51.7 (3.59) [38, 59]	50.8 (3.01) [41, 59]	53.5 (2.9) [44, 59]	49.5 (4.58) [35, 59]	51.2 (3.2) [40, 59]	53.8 (3.2) [23, 59]	***
Y	45.7 (11.65) [26, 59]	36.8 (11.14) [28, 59]	55.8 (3.83) [35, 59]	56.8 (2.27) [50, 59]	54 (4.79) [42, 59]	57.1 (2.06) [47, 59]	***
Z	51.6 (6.13) [32, 59]	53.7 (5.99) [39, 59]	52.3 (6.14) [32, 59]	50.6 (5.45) [44, 59]	53.3 (4.7) [41, 59]	48.5 (7.29) [30, 59]	***
**Mean**	**55.1 (2.60) [47, 58]**	**55.0 (3.00) [47, 59]**	**55.3 (3.17) [41, 59]**	**55.7 (2.94) [46, 59]**	**53.9 (3.08) [43, 57]**	**55.4 (2.78) [44, 58]**	

* *P* ≤ 0.05.

*** *P* ≤ 0.001.

Table 4 shows the individual mean, standard deviation, and range of tachycardia for each patient each night stratified by CPAP status. All the patients experienced tachycardia at night. Twenty-four patients’ tachycardia exhibited significant variability within each night (*P* < .0001) and 18 patients demonstrated significant variability across the 6 nights (*P*-values varied from <.05–<.0001). The grand means of the CPAP + cohort (119, SD 14.0) and the CPAP ‐ cohort (115, SD 4.0) were not significantly different (*P* = .57). Table S3, Supplemental Digital Content, http://links.lww.com/MD/N730 shows the coefficient of variation for the tachycardia for each patient each night and across nights.

**Table 4:** Mean (standard deviation) [range] of tachycardia for each patient who exhibited tachycardia across the 6 nights, blank spaces indicate nights where no heart rate value above 100 beats per minute values was recorded.

Patient	Night 1	Night 2	Night 3	Night 4	Night 5	Night 6	
CPAP+							
D	137.2 (43.7) [101, 242]	–	181 (39.19) [101, 197]	–	–	–	
E	104.6 (7.61) [101, 145]	101.9 (0.95) [101, 104]	102.3 (1.32) [101, 105]	102 (1) [101, 103]	103.6 (2.07) [101, 108]	102.5 (1.23) [101, 106]	***
F	115.1 (20.92) [101, 204]	–	115.4 (34.51) [104, 236]	131.2 (20.76) [107, 150]	–	122 (3.16) [113, 123]	
I	125.4 (40.09) [101, 224]	102.4 (2.3) [101, 110]	105 (14.92) [101, 230]	101 (0) [101, 101]	116.9 (9.37) [101, 126]	143.9 (8.22) [119, 154]	***
S	103.4 (1.99) [101, 108]	268.1 (73.7) [101, 296]	–	103 (1.79) [101, 112]	101 (NA) [101, 101]	101.8 (0.78) [101, 104]	***
U	104.7 (3.15) [101, 110]	112 (8.08) [101, 126]	108.5 (4.76) [101, 114]	110.4 (4.85) [101, 119]	107.7 (8.2) [101, 164]	104.3 (1.83) [101, 107]	***
X			131.7 (18.84) [104, 149]	103 (2.65) [101, 106]	105.7 (4.2) [101, 130]	110.8 (15.23) [101, 145]	***
**Mean**	**115 (19.6) [101, 172]**	**146 (21.3) [101, 159]**	**124 (18.9) [102, 172]**	**108 (5.17) [102, 115]**	**107 (5.96) [101, 126]**	**114 (5.08) [106, 123]**	
CPAP‐							
A	101.1 (0.36) [101, 102]	–	102.5 (1.66) [101, 106]	–	–	–	***
C	–	–	134.2 (42.83) [115, 224]	–	–	–	
G	101 (0) [101, 101]	105 (NA) [105, 105]	151 (0) [151, 151]	224 (0) [224, 224]		103.9 (1.48) [101, 105]	***
H	105.4 (3.32) [101, 114]	104 (2.7) [101, 112]	104 (2.75) [101, 111]	104 (3.47) [101, 119]	102.6 (1.42) [101, 108]	102.3 (1.63) [101, 106]	***
J	103 (3.18) [101, 115]	110.8 (18.73) [101, 163]	130.1 (47.86) [101, 240]	110.2 (29) [101, 250]	101.8 (1.1) [101, 103]	106.4 (18.48) [101, 280]	***
K	–	104.6 (1.86) [102, 108]	–	–	–	204 (NA) [204, 204]	***
L	126.7 (40.51) [101, 266]	116.6 (13.12) [101, 188]	112.9 (25.54) [101, 264]	107.7 (17.49) [101, 168]	101 (0) [101, 101]	106.2 (19.98) [101, 284]	***
M	101.7 (0.58) [101, 102]	107.1 (3.49) [101, 113]	114.6 (11.15) [101, 137]	–	–	103.6 (5.81) [101, 114]	***
N	–	120.2 (11.29) [103, 142]	–	–	180.5 (44.47) [101, 226]	–	***
O	–	–	–	–	–	–	
P	101.8 (1.26) [101, 104]	102.1 (1.18) [101, 107]	–	108.2 (21.15) [101, 230]	102.2 (1.22) [101, 104]	103.1 (4.39) [101, 222]	***
Q	–	–	104.3 (2.06) [101, 108]	102.1 (0.85) [101, 104]	–	111.1 (5.97) [101, 122]	***
R	–	106.3 (9.24) [101, 117]	–	–	–	–	
T	132.6 (7) [109, 138]	117.5 (2.12) [116, 119]	–	113.8 (14.82) [101, 150]	103.1 (1) [101, 104]	–	***
V	–	–	–	104.1 (1.62) [101, 107]	–	152.1 (61.84) [102, 256]	***
W	158.7 (52) [101, 206]	–	–	–	113.8 (20.21) [108, 178]	–	**
Y	102 (1.12) [101, 106]	102.7 (0.65) [101, 103]	102.6 (1.07) [101, 104]	102 (1.15) [101, 105]	–	102 (1.02) [101, 104]	
Z	–	–	104.5 (1.97) [101, 108]	–	–	–	
**Mean**	**113 (10.9) [102, 135]**	**109 (6.44) [103, 125]**	**116 (13.7) [107, 155]**	**120 (9.95) [115, 162]**	**115 (9.92) [102, 132]**	**119 (13.4) [111, 180]**	

** *P* ≤ 0.01.

*** *P* ≤ 0.001.

Table 5 shows the percentage of all readings collected for each patient each night (in one-second intervals) wherein patients had oxygen saturations <92%, heart rate <60 and >100 bpm, respectively. All patients exhibited pathologic oxygen desaturation and heart rates each night. We compared the mean percentage of abnormal oxygen saturation readings each night between the CPAP + cohort (48.00%, SD 3.69%) and the CPAP ‐ cohort (29.33%, SD 4.03%) and found that they were significantly different (*P* < .0001). In terms of the mean percentage of bradycardia, there was a significant difference between the CPAP + cohort (13.50%, SD 1.64%) and the CPAP ‐ cohort (11.50%, 1.22%), (*P* = .0398) but in terms of the mean percentage of tachycardia, there was not a significant difference between patients receiving CPAP and those not receiving CPAP (*P* = .41).

**Table 5:** Desaturation (oxygen saturation below 92%), bradycardia (heart rate values <60 beats per minute), and tachycardia (heart rate values >100 beats per minute) as percent of time for all values recorded for each patient over each of the 6 nights, grouped by CPAP prescription.

Patient	Night 1			Night 2			Night 3			Night 4			Night 5			Night 6		
	O2sat < 92	HR < 60	HR > 100	O2sat < 92	HR < 60	HR > 100	O2sat < 92	HR < 60	HR > 100	O2sat < 92	HR < 60	HR > 100	O2sat < 92	HR < 60	HR > 100	O2sat < 92	HR < 60	HR > 100
CPAP+																		
D	61%	1%	0%	80%	0%	0%	80%	0%	0%	72%	0%	0%	78%	0%	0%	68%	0%	0%
E	94%	0%	1%	61%	0%	0%	64%	0%	2%	73%	0%	0%	80%	0%	0%	78%	0%	0%
F	39%	90%	0%	38%	71%	0%	18%	90%	0%	15%	86%	0%	50%	93%	0%	9%	91%	0%
I	40%	0%	0%	71%	0%	0%	59%	0%	0%	35%	0%	0%	38%	0%	0%	7%	0%	0%
S	96%	1%	0%	74%	0%	0%	83%	0%	0%	62%	0%	3%	49%	0%	0%	91%	0%	0%
U	5%	1%	0%	36%	0%	2%	34%	0%	0%	8%	0%	1%	41%	0%	2%	54%	0%	0%
X	2%	19%	0%	1%	3%	0%	2%	0%	0%	19%	9%	0%	4%	6%	0%	33%	0%	0%
**Mean**	**48%**	**16%**	**0%**	**52%**	**11%**	**0%**	**49%**	**13%**	**0%**	**41%**	**14%**	**1%**	**49%**	**14%**	**0%**	**49%**	**13%**	**0%**
																		
CPAP-																		
A	5%	0%	0%	1%	0%	0%	1%	0%	1%	2%	1%	0%	2%	2%	0%	0%	1%	0%
B	50%	0%	0%	8%	13%	0%	13%	4%	0%	7%	0%	0%	13%	3%	0%	5%	1%	0%
C	92%	0%	0%	86%	0%	0%	91%	0%	0%	81%	0%	0%	84%	0%	0%	82%	0%	0%
G	15%	3%	0%	3%	5%	0%	11%	4%	0%	4%	9%	0%	15%	12%	0%	0%	13%	0%
H	13%	0%	1%	11%	0%	1%	20%	0%	1%	15%	0%	10%	6%	0%	1%	10%	0%	0%
J	100%	0%	1%	90%	0%	0%	99%	0%	1%	99%	0%	0%	99%	0%	0%	96%	0%	1%
K	79%	5%	0%	56%	0%	0%	52%	28%	0%	57%	13%	0%	48%	2%	0%	62%	2%	0%
L	81%	18%	0%	28%	6%	2%	12%	33%	1%	16%	16%	0%	24%	13%	0%	34%	9%	0%
M	2%	0%	0%	1%	0%	0%	3%	2%	0%	1%	0%	0%	3%	0%	0%	1%	3%	0%
N	63%	0%	0%	55%	0%	0%	51%	1%	0%	57%	0%	0%	29%	0%	2%	6%	0%	0%
O	9%	0%	0%	7%	0%	0%	16%	0%	0%	1%	0%	0%	12%	0%	0%	10%	0%	0%
P	29%	0%	0%	52%	0%	1%	71%	0%	0%	25%	0%	1%	3%	0%	1%	20%	0%	30%
Q	17%	0%	0%	11%	0%	0%	24%	0%	1%	8%	0%	0%	15%	0%	0%	21%	0%	1%
R	4%	65%	0%	2%	97%	0%	3%	82%	0%	4%	76%	0%	10%	86%	0%	3%	69%	0%
T	35%	1%	0%	54%	4%	0%	15%	1%	0%	64%	1%	0%	63%	1%	0%	23%	9%	0%
V	2%	19%	0%	2%	26%	0%	1%	4%	0%	2%	1%	0%	3%	1%	0%	1%	3%	0%
W	23%	87%	0%	35%	98%	0%	13%	93%	0%	4%	96%	0%	11%	93%	0%	7%	84%	0%
Y	39%	4%	0%	40%	2%	0%	57%	2%	0%	44%	1%	0%	56%	2%	0%	87%	1%	0%
Z	39%	1%	0%	18%	1%	0%	18%	1%	0%	22%	0%	0%	11%	0%	0%	18%	2%	0%
**Mean**	**37%**	**11%**	**0%**	**29%**	**13%**	**0%**	**30%**	**13%**	**0%**	**27%**	**11%**	**1%**	**27%**	**11%**	**0%**	**26%**	**10%**	**2%**

**Figure 1.**
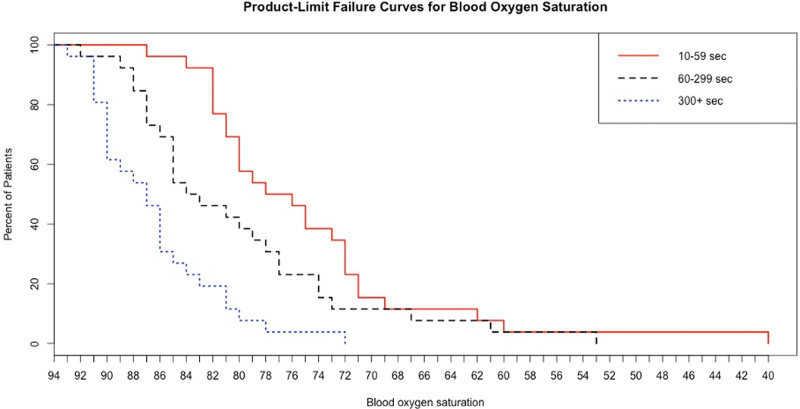
Kaplan–Meier desaturation plots for 3 duration intervals, 10 to 59 seconds, 60 to 299 seconds, and 300+ seconds. For each curve (a) cohort of patients defined by their duration of desaturation), the *x*-axis represents the nadir oxygen desaturation for that cohort, and the *y*-axis represents the percentage of patients experiencing those desaturations.

Figure 1 shows the Kaplan–Meier desaturation plots for 3 duration intervals, 10 to 59 seconds, 60 to 299 seconds, and 300+ seconds. For each curve (a cohort of patients defined by their duration of desaturation), the *x*-axis represents the nadir oxygen desaturation for that cohort, and the *y*-axis represents the percentage of patients experiencing those desaturations. Two of the 3 desaturation duration cohort comparisons are significantly different: 10 to 59 second versus 60 to 299 (*P* = .0580), 60 to 299 versus 300 + (*P* = .0040), and 10 to 59 versus 300+ (*P* < .0001).

**Figure 2.**
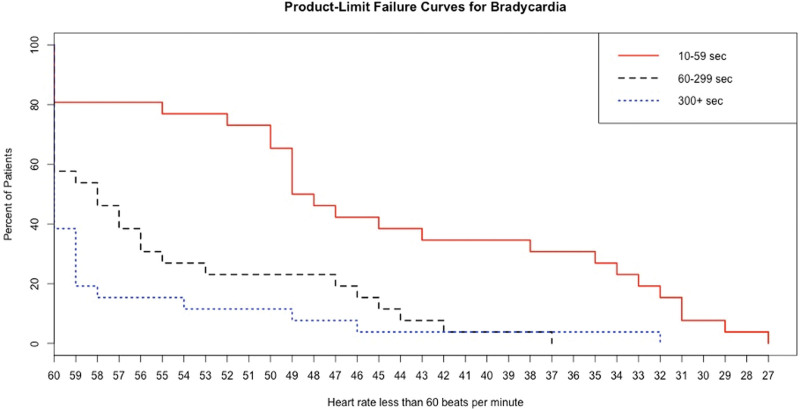
Kaplan–Meier bradycardia plots for 3 duration intervals, 10 to 59 seconds, 60 to 299 seconds, and 300+ seconds. For each curve (a) cohort of patients defined by their duration of bradycardia), the *x*-axis represents the rate for that cohort, and the *y*-axis represents the percentage of patients experiencing bradycardia.

Figure 2 shows the Kaplan–Meier bradycardia plots for 3 duration intervals, 10 to 59 seconds, 60 to 299 seconds, and 300+ seconds. For each curve (a cohort of patients defined by their duration of bradycardia), the *x*-axis represents the rate for that cohort, and the *y*-axis represents the percentage of patients experiencing bradycardia. Two of the 3 bradycardia duration cohort comparisons are significantly different: 10 to 59 second versus 60 to 299 (*P* = .0005), 60 to 299 versus 300+ (NS), and 10 to 59 versus 300+ (*P* < .0001).

**Figure 3.**
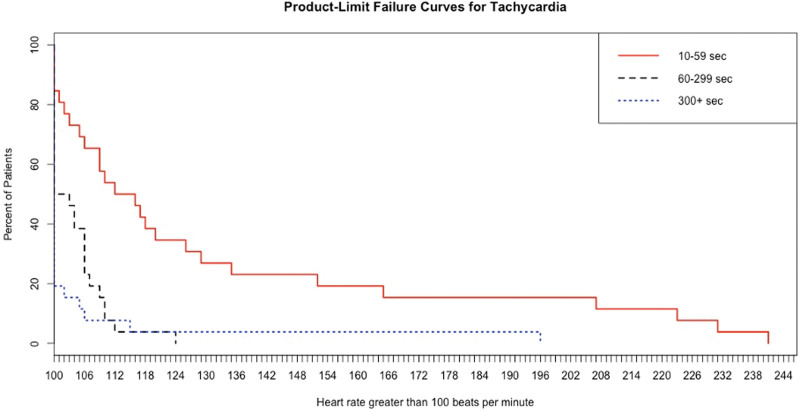
Kaplan–Meier tachycardia plots for 3 duration intervals, 10 to 59 seconds, 60 to 299 seconds, and 300+ seconds. For each curve (a) cohort of patients defined by their duration of tachycardia), the *x*-axis represents the rate for that cohort, and the *y*-axis represents the percentage of patients experiencing tachycardia.

Figure 3 shows the Kaplan–Meier tachycardia plots for 3 duration intervals, 10 to 59 seconds, 60 to 299 seconds, and 300+ seconds. For each curve (a cohort of patients defined by their duration of tachycardia), the *x*-axis represents the rate for that cohort, and the *y*-axis represents the percentage of patients experiencing tachycardia. Two of the 3 tachycardia duration cohort comparisons are significantly different 10 to 59 second versus 60 to 299 (*P* = .0001), 60 to 299 versus 300+ (NS), and 10 to 59 versus 300+ (*P* < .0001).

**Figure 4.**
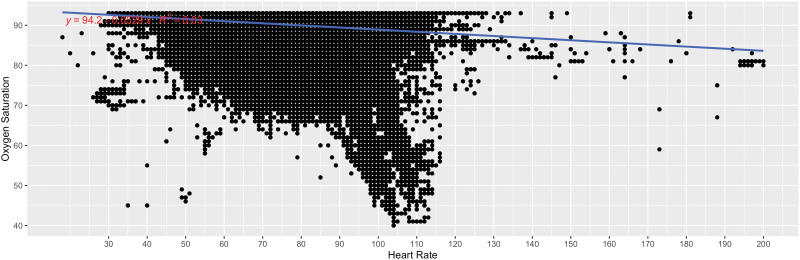
Correlation plot for each oxygen saturation value <94% and its associated heart rate reading for all heart rates and for all patients over all nights with linear regression and *R*^2^ for the association. For oxygen-heart rate pairs, 2,688,147 of 4,370,336 (pairs, 61.5)% of oxygen saturation values were <94%. We removed 138 additional reading pairs with associated heart rates >220 as extreme outliers, resulting in 2,688,009 of 4,370,336 pairs available for analysis.

Figure 4 shows the correlation plot for each oxygen saturation value <94% and its associated heart rate reading for all heart rates and for all patients over all nights with linear regression and *R*^2^ for the association. For oxygen-heart rate pairs, 2,688,147 of 4,370,336 (pairs, 61.5)% of oxygen saturation values were <94%. We removed 138 additional reading pairs with associated heart rates >220 as extreme outliers, resulting in 2,688,009 of 4,370,336 pairs available for analysis. Overall, there was no association between low oxygen saturation and heart rate (*R*^2^ = 0.03).

## 4. Discussion

We have previously shown that heart failure patients can reliably use wearable devices at home at night for 6 consecutive nights, and we have also shown that the information collected by these devices can be displayed in a way the clinicians find useful in their management of heart failure patients.^[32,33]^ We now report that wearable devices can capture clinically important desaturations and arrhythmias at night. We found that all the heart failure patients experienced grave pathological desaturations and arrhythmias within and across nights and that these clinical events were captured by wearable devices. These desaturations and arrhythmias may be clinically important, and they may be one of the reasons why patients decompensate and present unexpectedly to the Emergency Department in the middle of the night. Our results suggest that in addition to clinic assessments and patient reports, 6-night studies can provide important clinical information that may assist in the management of heart failure patients.

It is known that heart failure patients exhibit sleep disordered breathing,^[31,38–41]^ which can be associated with hypoxia. It is also known that sleep disordered breathing can lead to bradyarrhythmias, and sympatho-excitation favoring ventricular ectopy^[42]^ and that obstructive sleep apnea in hospitalized patients was associated with tachyarrhythmias and bradyarrhythmias in heart failure patient with a preserved ejection fraction.^[43–49]^ A 24-hour ambulatory pulse oximetry study of 10 heart failure patients found significant decreases in oxygen saturation, and long and deep nadirs of desaturation.^[50]^ Moreover, analysis of electrocardiogram data over several nights shows cyclic variation in heart rate associated with sleep apnea.^[51]^ Wearable finger pulse oximetry in patients with chronic obstructive pulmonary disease has shown variability of oxygen saturation and demonstrated increased utility over spot check methods alone.^[52]^

It has long been recognized that heart failure patients are not very good at self-reporting their symptoms.^[15]^ In fact, many patients only present to the Emergency Department because of relatives or referral from clinic,^[7]^ where almost all are admitted.^[53]^ Furthermore, there is not a strong relationship between patients’ objective sleep disordered breathing and their self-reported daytime symptoms or sleep.^[54,55]^ Thus, objective nocturnal data over 6 nights may be clinically important.

Wearable devices are being used to measure heart rates and rhythms.^[17]^ Telemedicine has assessed their ability to provide information that can be used for patient management.^[32,33,44–49]^ Unfortunately, these telemedicine studies did not report nocturnal oxygen saturations and heart rates.

We found significant desaturation and arrhythmia differences between patients receiving CPAP and those not receiving CPAP. A recent meta-analysis of CPAP found that it improves sleep, cardiovascular outcomes, and quality-of-life at 3 months, but it is unclear whether it confers longer-term cardiovascular and mortality benefits.^[56]^

Decompensations at night are related to patients’ activities during the day. Nighttime can be thought of as the final common pathway for the healthy and unhealthy events of the day. Thus, night is an excellent time to assess a heart failure patient’s clinical status. In support of the nocturnal collection of clinical data, a recent study found that nocturnal blood pressure readings were significantly more associated with cardiovascular events than daytime readings.^[57]^ Furthermore, night is an efficient time to acquire objective information because, during the day, patients may forget to wear the device, the device may not always be in direct contact with the skin, and their activities of daily living may interfere with their wearing the device.^[14,17]^

We believe that there were a sufficient number of heart failure patients to demonstrate the clinically important intra-patient and inter-patient oxygen desaturations and arrhythmias occurring over 6 nights. The observed CPAP benefit may be greater than that demonstrated in this study because the patients requiring CPAP may have had more severe disease. In terms of arrhythmias, some arrhythmias may have been attenuated in patients with implanted cardiac devices.

These results raise important safety and quality of life issues. Patients experiencing pathologic desaturations and arrhythmias may be at risk for worsening heart failure and they may be at a higher risk of death. In addition, their sleep quality is affected by their desaturations and arrhythmias, resulting in decreased quality of life.^[14]^ Managing patients based on their nocturnal clinical pathology may improve their quality of life and extent their lives.

Heart failure presents complex clinical management challenges. The better our objective information regarding these patients’ clinical status, the better we can manage them. Wearable devices can assist in patient management by providing clinically actionable information, and they can they be used to regularly assess the clinical status of heart failure patients at night over 6 successive nights, perhaps every 6 months. Furthermore, we suggest that when starting or stopping a treatment, including CPAP, a 6-night study be conducted. We also suggest that this clinical information be displayed in a way that clinicians can use to manage their heart failure patients, preferably, as part of a clinical decision support system.^[33]^

In conclusion, we found that heart failure patients experience clinically important and highly variable desaturations and arrhythmias over several nights and that these clinical events can be captured by wearable devices. Because these desaturations and arrhythmias may be clinically important, we should not rely on a single-night sleep study or a 24-hour heart rate monitor because they will not capture the full extent of patients’ oxygenation and heart rate decompensations. Our study underscores the efficacy of wearable devices in medical research. These devices, despite their compact form, can capture critical nocturnal data. A study spanning just 6 nights revealed a wealth of clinical insights, invaluable for tailoring patient management strategies. It is noteworthy that our endeavor is novel, marking the first comprehensive examination of nighttime oxygen levels and heart rates in heart failure patients residing at home. To our knowledge, this is the first ecologically valid moment-by-moment synchronous nocturnal study of oxygen saturations and heart rates at night for 6 nights in home-dwelling heart failure patients.

## Author contributions

**Conceptualization:** Cubby L. Gardner, Harry B. Burke.

**Data curation:** Cubby L. Gardner.

**Formal analysis:** Cubby L. Gardner, Harry B. Burke.

**Funding acquisition:** Cubby L. Gardner, Harry B. Burke.

**Investigation:** Cubby L. Gardner.

**Methodology:** Cubby L. Gardner.

**Project administration:** Cubby L. Gardner, Harry B. Burke.

**Resources:** Cubby L. Gardner.

**Software:** Cubby L. Gardner.

**Supervision:** Cubby L. Gardner, Harry B. Burke.

**Validation:** Cubby L. Gardner, Harry B. Burke.

**Visualization:** Cubby L. Gardner.

**Writing – original draft:** Cubby L. Gardner.

**Writing – review & editing:** Cubby L. Gardner, Harry B. Burke.

## Supplementary Material


