# Supplementary material for: Individual heart failure patient variability in nocturnal hypoxia and arrhythmias
Source: Medicine (Baltimore). 2024 Oct 11;103(41):e40083. doi: 10.1097/MD.0000000000040083 (PMC11479525; doi:10.1097/MD.0000000000040083)
Supplement: Supplementary file 1 [file medi-103-e40083-s001.docx]

Supplemental Table 1. Coefficient of variation of desaturation for each patient across the six nights.

| Patient | Night 1 | Night 2 | Night 3 | Night 4 | Night 5 | Night 6 | Mean (SD) |
| --- | --- | --- | --- | --- | --- | --- | --- |
| CPAP+ |  |  |  |  |  |  |  |
| D | 2.88 | 2.75 | 2.74 | 3.46 | 2.74 | 3.44 | 3.00 (0.35) |
| E | 2.00 | 2.26 | 2.53 | 1.60 | 2.03 | 2.38 | 2.13 (0.33) |
| F | 2.39 | 2.36 | 1.89 | 1.96 | 3.39 | 1.82 | 2.30 (0.59) |
| I | 2.59 | 2.47 | 2.24 | 1.68 | 2.27 | 1.76 | 2.17 (0.37) |
| S | 2.30 | 2.15 | 3.50 | 2.81 | 3.36 | 2.31 | 2.74 (0.58) |
| U | 1.54 | 1.90 | 1.89 | 1.91 | 2.01 | 2.04 | 1.88 (0.18) |
| X | 1.19 | 1.20 | 1.02 | 1.88 | 1.55 | 1.89 | 1.46 (0.38) |
| **Mean (SD)** | **2.13 (0.59)** | **2.16 (0.50)** | **2.26 (0.78)** | **2.19 (0.69)** | **2.48 (0.71)** | **2.23 (0.58)** | **2.24 (0.51)** |
| CPAP- |  |  |  |  |  |  |  |
| A | 1.07 | 1.23 | 1.17 | 1.45 | 1.16 | 1.19 | 1.21 (0.13) |
| B | 2.27 | 1.99 | 1.99 | 1.73 | 1.93 | 1.52 | 1.90 (0.26) |
| C | 2.68 | 3.31 | 3.53 | 3.09 | 2.77 | 3.77 | 3.19 (0.43) |
| G | 1.60 | 1.55 | 1.52 | 1.45 | 1.60 | 1.17 | 1.48 (0.16) |
| H | 1.90 | 1.81 | 1.92 | 1.87 | 1.72 | 1.62 | 1.81 (0.12) |
| J | 5.02 | 6.90 | 4.57 | 4.77 | 4.10 | 5.46 | 5.14 (0.98) |
| K | 3.56 | 4.41 | 3.83 | 3.23 | 2.69 | 2.83 | 3.42 (0.65) |
| L | 4.81 | 4.15 | 4.37 | 3.45 | 3.53 | 3.55 | 3.98 (0.55) |
| M | 1.18 | 1.48 | 1.32 | 1.16 | 1.51 | 1.39 | 1.34 (0.15) |
| N | 4.78 | 3.95 | 4.06 | 3.19 | 4.38 | 2.00 | 3.73 (1.00) |
| O | 1.92 | 1.69 | 1.66 | 1.57 | 2.02 | 1.99 | 1.81 (0.19) |
| P | 2.77 | 5.38 | 3.62 | 5.07 | 2.76 | 6.53 | 4.36 (1.54) |
| Q | 3.05 | 2.48 | 3.85 | 2.16 | 2.78 | 3.08 | 2.90 (0.58) |
| R | 1.84 | 1.50 | 1.74 | 2.03 | 2.19 | 2.19 | 1.92 (0.27) |
| T | 2.06 | 2.15 | 1.75 | 1.67 | 1.86 | 1.98 | 1.91 (0.18) |
| V | 1.40 | 1.29 | 1.15 | 1.34 | 1.18 | 1.60 | 1.33 (0.16) |
| W | 3.14 | 3.16 | 2.12 | 2.10 | 3.55 | 2.17 | 2.71 (0.65) |
| Y | 3.91 | 3.67 | 2.07 | 1.86 | 2.04 | 2.27 | 2.64 (0.91) |
| Z | 2.97 | 2.87 | 2.49 | 2.59 | 3.25 | 2.66 | 2.81 (0.28) |
| **Mean (SD)** | **2.73 (1.23)** | **2.89 (1.55)** | **2.56 (1.17)** | **2.41 (1.12)** | **2.47 (0.95)** | **2.56 (1.42)** | **2.61 (1.13)** |
| Standard deviation (SD) | | | | | | | |

Supplemental Table 2. Coefficient of variation of bradycardia for each patient across the six nights.

| Patient | Night 1 | Night 2 | Night 3 | Night 4 | Night 5 | Night 6 | Mean (SD) |
| --- | --- | --- | --- | --- | --- | --- | --- |
| CPAP+ |  |  |  |  |  |  |  |
| D | 2.23 | .. | .. | 23.60 | 17.25 | 18.85 | 15.48 (9.24) |
| E | .. | .. | 19.09 | .. | .. | .. | 19.09 (0.00) |
| F | 4.16 | 3.72 | 5.09 | 5.20 | 5.79 | 7.52 | 5.25 (1.34) |
| I | .. | .. | .. | 0.00 | 23.17 | 24.28 | 15.82 (13.71) |
| S | 11.45 | 6.01 | 4.81 | 7.60 | 12.11 | 6.63 | 8.10 (3.00) |
| U | 2.10 | 0.00 | .. | .. | 28.71 | 4.72 | 8.88 (13.36) |
| X | 2.98 | 1.93 | 1.02 | 2.53 | 3.30 | 3.36 | 2.52 (0.91) |
| **Mean (SD)** | **4.58 (3.92)** | **2.92 (2.56)** | **7.50 (7.94)** | **7.79 (9.29)** | **15.06 (9.90)** | **10.89 (8.57)** | **10.73 (6.14)** |
| CPAP- |  |  |  |  |  |  |  |
| A | .. | 1.68 | .. | 5.18 | 6.97 | 1.17 | 3.75 (2.79) |
| B | 0.80 | 2.79 | 2.72 | 1.44 | 6.00 | 2.14 | 2.65 (1.81) |
| C |  | .. | .. | .. | .. | 17.43 | 17.43 (0.00) |
| G | 12.40 | 1.52 | 5.13 | 6.08 | 4.83 | 1.01 | 5.16 (4.10) |
| H | .. | .. | .. | .. | .. | .. | .. |
| J | .. | .. | 20.91 | 6.64 | 7.81 | 12.58 | 11.98 (6.48) |
| K | 2.27 | 2.67 | 2.86 | 1.81 | 4.08 | 6.07 | 3.29 (1.56) |
| L | 3.84 | 9.86 | 4.92 | 4.30 | 3.98 | 4.50 | 5.23 (2.30) |
| M | 0.31 | 1.56 | 2.27 | 1.92 | 2.47 | 3.68 | 2.04 (1.11) |
| N | 0.00 | 11.5 | 4.28 | 10.21 | 16.36 | .. | 8.47 (6.40) |
| O | 1.52 | 0.00 | .. | 8.42 | 2.34 | 1.90 | 2.84 (3.24) |
| P | .. | .. | .. | .. | .. | .. | .. |
| Q | 0.00 | .. | 5.17 | .. | .. | .. | 2.58 (3.66) |
| R | 2.76 | 2.55 | 3.48 | 2.77 | 2.99 | 2.45 | 2.83 (0.37) |
| T | 0.88 | 1.50 | 6.66 | 6.69 | 2.68 | 1.20 | 3.27 (2.71) |
| V | 2.30 | 3.70 | 2.71 | 1.79 | 1.64 | 2.70 | 2.47 (0.75) |
| W | 6.94 | 5.92 | 5.41 | 9.24 | 6.25 | 5.94 | 6.62 (1.38) |
| Y | 25.51 | 30.29 | 6.85 | 3.99 | 8.87 | 3.60 | 13.19 (11.66) |
| Z | 11.88 | 11.14 | 11.73 | 10.79 | 8.83 | 15.01 | 11.56 (2.01) |
| **Mean (SD)** | **5.10 (7.16)** | **6.19 (7.91)** | **6.08 (4.91)** | **5.42 (3.20)** | **5.74 (3.79)** | **5.43 (5.29)** | **6.20 (4.66)** |
| Standard deviation (SD) | | | | | | | |

Supplemental Table 3. Coefficient of variation of tachycardia for each patient across the six nights.

| Patient | Night 1 | | Night 2 | | Night 3 | | Night 4 | | Night 5 | | Night 6 | | Mean (SD) | |
| --- | --- | --- | --- | --- | --- | --- | --- | --- | --- | --- | --- | --- | --- | --- |
| CPAP+ |  | |  | |  | |  | |  | |  | |  | |
| D | 31.84 | | .. | | 21.65 | | .. | | .. | | .. | | 26.74 (7.21) | |
| E | 7.28 | | 0.94 | | 1.29 | | 0.98 | | 2.00 | | 1.20 | | 2.28 (2.48) | |
| F | 18.18 | | .. | | 29.90 | | 15.83 | | .. | | 2.59 | | 16.62 (11.20) | |
| I | 31.98 | | 2.25 | | 14.21 | | 0.00 | | 8.01 | | 5.71 | | 10.36 (11.68) | |
| S | 1.93 | | 27.49 | | .. | | 1.74 | | .. | | 0.77 | | 7.98 (13.01) | |
| U | 3.01 | | 7.22 | | 4.39 | | 4.39 | | 7.61 | | 1.76 | | 4.73 (2.30) | |
| X |  | |  | | 14.31 | | 2.57 | | 3.97 | | 13.74 | | 8.65 (6.24) | |
| **Mean (SD)** | | **15.70 (13.81)** | | **9.48 (12.31)** | | **14.29 (10.62)** | | **4.25 (5.87)** | | **5.40 (2.90)** | | **4.30 (4.95)** | | **11.055 (8.26)** |
| CPAP- |  | |  | |  | |  | |  | |  | |  | |
| A | 0.35 | | .. | | 1.62 | | .. | | .. | | .. | | 0.99 (0.90) | |
| B | .. | | .. | | .. | | .. | | .. | | .. | | .. | |
| C | .. | | .. | | 31.91 | | .. | | .. | | .. | | 31.91 (0.00) | |
| G | 0.00 | | .. | | 0.00 | | 0.00 | | .. | | 1.42 | | 0.36 (0.71) | |
| H | 3.15 | | 2.59 | | 2.64 | | 3.33 | | 1.38 | | 1.60 | | 2.45 (0.80) | |
| J | 3.09 | | 16.89 | | 36.78 | | 26.32 | | 1.08 | | 17.38 | | 16.92 (13.60) | |
| K | .. | | 1.78 | | .. | | .. | | .. | | .. | | 1.78 (0.00) | |
| L | 31.96 | | 11.25 | | 22.61 | | 16.24 | | 0.00 | | 18.82 | | 16.81 (10.78) | |
| M | 0.57 | | 3.26 | | 9.72 | | .. | | .. | | 5.61 | | 4.79 (3.88) | |
| N | .. | | 9.39 | | .. | | .. | | 24.64 | | .. | | 17.02 (10.78) | |
| O | .. | | .. | | .. | | .. | | .. | | .. | | .. | |
| P | 1.24 | | 1.16 | | .. | | 19.55 | | 1.19 | | 4.26 | | 5.48 (7.98) | |
| Q | .. | | .. | | 1.97 | | 0.84 | | .. | | 5.37 | | 2.73 (2.36) | |
| R | .. | | 8.69 | | .. | | .. | | .. | | .. | | 8.69 (0.00) | |
| T | 5.28 | | 1.81 | | .. | | 13.02 | | 0.97 | | .. | | 5.27 (5.49) | |
| V | .. | | .. | | .. | | 1.55 | | .. | | 40.66 | | 21.1 (27.65) | |
| W | 32.76 | | .. | | .. | | .. | | 17.75 | | .. | | 25.25 (10.61) | |
| Y | 1.10 | | 0.63 | | 1.05 | | 1.13 | | .. | | 1.00 | | 0.98 (0.20) | |
| Z | .. | | .. | | 1.89 | | .. | | .. | | .. | | 1.89 (0.00) | |
| **Mean (SD)** | **9.75 (12.97)** | | **5.75 (5.49)** | | **11.02 (14.07)** | | **9.11 (9.85)** | | **6.72 (10.10)** | | **10.68 (13.07)** | | **9.67 (9.84)** | |
| Standard deviation (SD) | | | | | | | | | | | | | | |
